# Impact of multi-professional medication reviews (MPMRs) on medication prescriptions in psychiatry

**DOI:** 10.1371/journal.pone.0358539

**Published:** 2026-09-21

**Authors:** Matthieu Lebrat, Ahava Cohen, Rachel Megard, Nicolas Franck, Claude Dussart, Marion Perin-Dureau

**Affiliations:** 1 Pharmacie et Stérilisation Centrales, Hospices Civils de Lyon, Lyon, France; 2 Université Lyon 1, P2S, UR 4129, Lyon, France; 3 Université Lyon 1, Lyon, France; 4 Centre Ressource de Réhabilitation psychosociale, Pôle Centre Rive Gauche, Le Vinatier Hospital, Bron, France; 5 Pôle Ouest, Le Vinatier Hospital, Bron, France; 6 UMR 5229 CNRS, Le Vinatier Hospital, Bron, France; Karolinska Institutet, SWEDEN

## Abstract

**Purpose:**

Multi-professional medication reviews (MPMRs) are structured, collaborative processes designed to optimize medication management and promote evidence-based practice. Yet, their impact in psychiatric settings remains understudied. This study aimed to retrospectively assess the impact of hospital-based MPMRs on medication prescriptions in psychiatric inpatients.

**Methods:**

An observational, retrospective, pre-post study in four psychiatric wards of a French university hospital was conducted. Participants had two hospital stays: one before and one after MPMR implementation (August 2017–December 2023). Data collection was performed from electronic medical records. The primary outcome was the change in the number of prescribed medications between admission and discharge, compared pre- and post-MPMR implementation. Secondary outcomes included changes in specific drug classes. Data were analyzed using paired tests and mixed linear models (MLM), adjusting for age, sex, hospitalization duration, and ward.

**Results:**

MPMR implementation was associated with a significant reduction in the total number of prescribed medications (−1.1 drugs, p = 0.044 or 0.001 with a MLM, Cohen’s *d* = 0.18). Significant decreases were observed in overall psychotropic medications (−1.5, p < 0.001), benzodiazepines (−0.8, p < 0.001), antipsychotics (−0.3, p < 0.05), anticholinergics (−0.7, p ≤ 0.05), and laxatives (−0.2, p ≤ 0.05). No significant changes were found for antidepressants, mood stabilizers, or analgesics. Sex differences were noted in overall psychotropic deprescribing, with men showing greater reduction.

**Conclusion:**

MPMRs in psychiatric inpatient settings may improve prescribing practices, particularly by reducing high-risk and potentially inappropriate medications. While the overall reduction in total medications was modest, targeted decreases in psychotropics and anticholinergics suggest clinically meaningful benefits for patient safety and medication appropriateness. Further research with randomized designs is warranted to confirm these findings and explore long-term outcomes.

## Introduction

Multi-professional medication review (MPMR) is a structured process designed to optimize medication management. It involves collaboration among various healthcare professionals involved in the patient’s care pathway, who jointly review the patient’s prescriptions to reassess the benefit–risk balance of each medication.

According to the Cochrane Library, a medication review can be defined as a comprehensive evaluation conducted by healthcare professionals to optimize medication usage and improve health outcomes [1]. This process enables professionals to identify, prevent, and address drug-related issues, which can range from prescribing errors or the need for alternative pharmacotherapeutic options to monitoring issues. Multi-professional approaches on medication management are recommended globally in many health systems to improve safe medication use [2–4]. Psychiatric practice is inherently interdisciplinary, requiring collaboration among various healthcare professionals to deliver optimal patient care [5].

Among therapeutic approaches, pharmacotherapy plays a central role in the treatment of mental illness. However, the use of psychotropic medications presents significant challenges for clinicians, owing to their complex mechanisms of action, the risk of drug–drug interactions, the need for extensive therapeutic monitoring, and the potential for medication non-adherence [6–8]. Furthermore, individuals with mental health disorders often require medications from multiple classes to manage both their psychiatric conditions and somatic comorbidities, thereby amplifying the risks associated with polypharmacy.

Numerous studies have identified potentially inappropriate medications in discharge prescriptions from psychiatric wards, particularly among older adults [9–11]. In addition, a systematic review of English, German, and French databases (1999–2023) reported that the prevalence of drug-related problems (DRPs) ranged from 0.32 to 9.48 per psychiatric patient, with most DRPs attributed to prescribing errors (1.91 per patient). Drug–drug interactions were the most common subtype (0.77 per patient). In their inpatient setting, the authors highlighted the importance of a well-structured medication reconciliation as a crucial first step in identifying DRPs and improving overall medication management [12].

The Pharmaceutical Care Network Europe (PCNE) defines medication reconciliation as a process, or a set of activities, aimed at obtaining an accurate and complete understanding of a patient’s intended and actual medication use during all transitions of care. This process does not necessarily involve identifying DRPs beyond unintended medication discrepancies and can therefore be considered the initial step in a broader medication review [13].

MPMRs have already demonstrated their effectiveness across various outcomes and medical specialties, particularly in oncology and geriatrics [14,15]. For instance, in care homes, MPMRs improved medication appropriateness in a cluster randomized controlled trial [16]. In internal medicine wards, structured MPMRs identified an average of 3 drug-related problems per patient, with 80% of them addressed through subsequent interventions [17].

Medication reviews typically involve several sequential steps performed by different healthcare professionals. The pharmacist first identifies potential DRPs, followed by consultation with a nurse for patient-specific information, and finally, the physician reviews the findings and may modify the prescription if necessary. However, existing evidence suggests that these reviews may be more effective when conducted from a genuinely multidisciplinary perspective [18]. Therein lies the advantage of the MPMR model assessed in our study, with its simultaneous, collaborative approach. In our setting, a psychiatric hospital ward, dedicated time is regularly allocated for joint, multi-professional analysis of each patient’s medication regimen.

Increasing evidence supports the integration of clinical pharmacists within multidisciplinary mental health teams, where their involvement improves the identification of drug-related problems, medication reconciliation, prescribing quality, and medication safety [19,20]. In our MPMR process, this expertise is integrated into a structured multidisciplinary discussion during which the benefit-risk balance of every medication prescribed to every inpatient is systematically reassessed. The combined expertise of the multidisciplinary team, which includes, among other professionals, a clinical pharmacist, is then leveraged to optimize the efficiency and clinical impact of this intervention.

Case conferences share similarities with MPMRs, as both employ a multidisciplinary approach to addressing potentially inappropriate medication [21–23]. However, they are typically limited in duration and scope, focusing on a single patient or a specific issue. In contrast, MPMR encompasses all prescriptions for all patients, analyzing every component of the treatment regimen, a systematic practice that remains less explored in the literature. Earlier systematic reviews did not identify interventions comparable to our model [24]. Likewise, a 2023 Cochrane review of medication reviews conducted by healthcare professionals for hospitalized adults did not identify similar interventions [1]. Although one study included a MPMR, it formed part of a broader intervention package rather than a focused, systematic review of all ward prescriptions [25]. The study most closely resembling our approach was a multidisciplinary medication review conducted in nursing homes, which involved pharmacists, physicians, and nurses. This intervention resulted in improved drug therapy, enhanced monitoring of side effects and drug interactions, and reduced medication costs [26].

Recent evidence has expanded the literature on multidisciplinary medication management in psychiatric settings. Several studies have demonstrated that structured interdisciplinary approaches involving pharmacists improve medication safety during transitions of care and reduce medication-related problems. In a quasi-experimental study conducted in a psychogeriatric unit, interdisciplinary medication reconciliation at discharge significantly reduced reconciliation errors from 55.2% to 37% [27]. Likewise, a quality-improvement project in an inpatient psychiatry unit identified numerous prescribing errors, highlighting the value of systematic multidisciplinary review [28], while a pilot study in a psychiatric emergency department demonstrated significant reductions in medication discrepancies following pharmacist-supported medication reconciliation [29].

Nevertheless, these interventions mainly focus on medication reconciliation at transitions of care, discharge optimisation, or targeted pharmacist-led reviews. While multidisciplinary medication optimisation in psychiatric settings has been increasingly described in recent years, this ward-wide multidisciplinary medication review model, in which all prescriptions for all psychiatric inpatients are jointly reassessed during routine clinical practice, has received comparatively little attention. This distinction underpins the rationale for the present study. Therefore, the aim of the present study was to retrospectively assess the impact of hospital-based MPMRs on medication prescriptions in psychiatric inpatients.

## Methods

### Study design and setting

Our study is an observational, retrospective, single-center, pre-post study. The 4 study wards in the present monocentric study were psychiatric departments of a university hospital specialized in mental health. Patients were hospitalized in these wards for various mental health conditions.

### Study population

All participants included in the study were adults hospitalized at the Centre Hospitalier Le Vinatier (Bron, France) between 08/01/2017 and 12/31/2023. To be eligible, patients had to have experienced at least two hospital stays: one before the implementation of MPMRs (pre-intervention) and one after, during which they received at least one MPMR (post-intervention). MPMRs were implemented on 1/1/2020.

Patients were identified using available data covering the 29 months before and the 48 months after the implementation of the MPMR program (from August 1, 2017, to December 31, 2023). The hospital stay occurring immediately prior to the implementation of the MPMR was defined as the pre-intervention stay. To ensure data independence, only the first hospital admission during which a MPMR was conducted (post-intervention) was included in the analysis.

The use of each patient’s pre-intervention stay as a comparator aimed to minimize potential confounding factors, such as inter-individual variability and differences in prescribing practices between patients. However, given the fluctuating nature of psychiatric disorders and the evolution of patients’ clinical status over time, changes in prescribing patterns may also reflect variations in disease presentation, treatment response, or illness progression, rather than the effect of the intervention alone. To minimize potential confounding due to ward-specific differences in prescribing practices or care organization, only patients who were hospitalized in the same psychiatric ward before and after the implementation of the MPMR were included. This restriction aimed to reduce selection bias and ensure that any observed changes in prescribing patterns could be more confidently attributed to the intervention itself.

**Fig 1:**
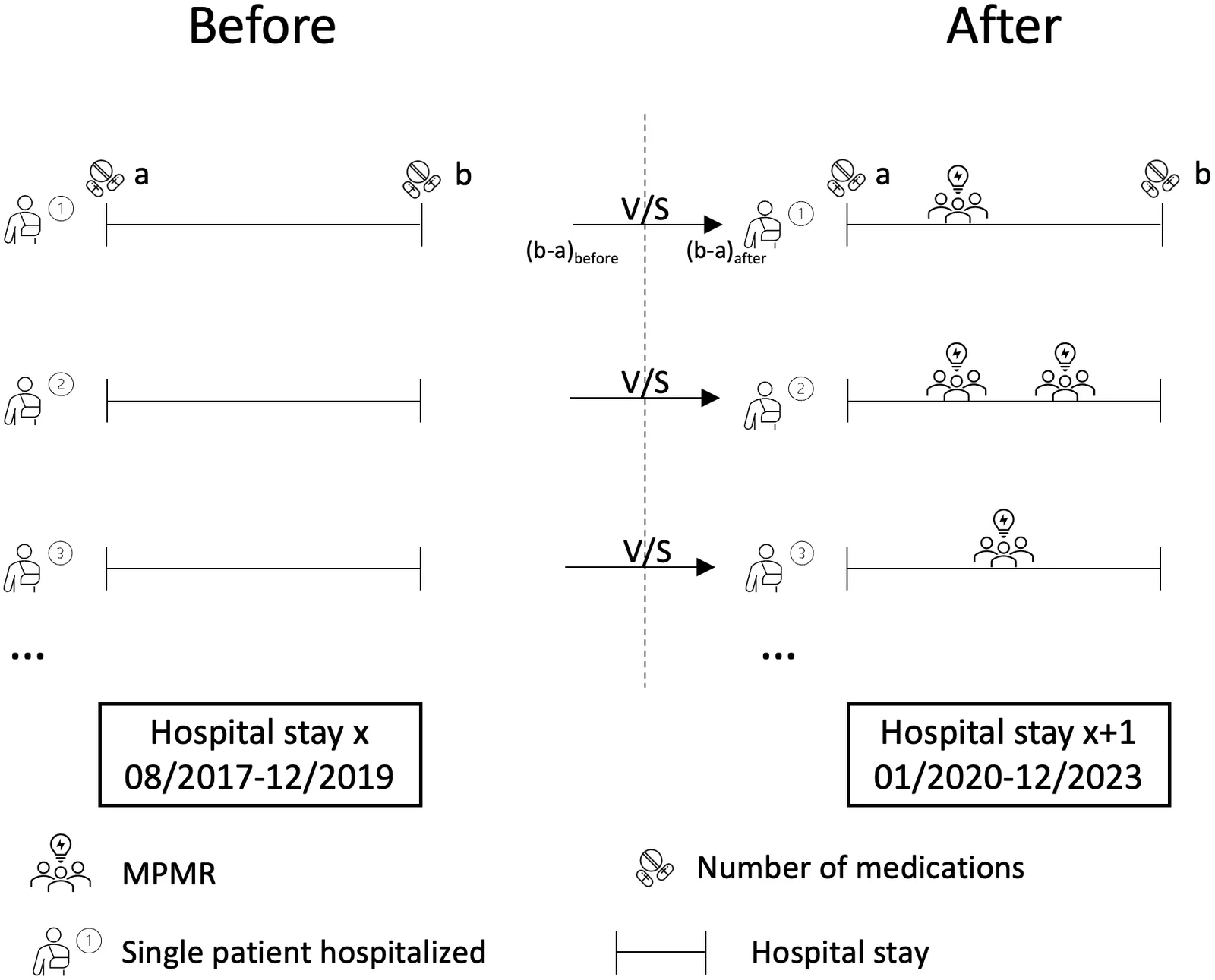
Study design and population. V/S: versus. MPMR: Multi-professional medication review.

Fig 1 summarizes the study design and the study population. The only exclusion criterion was patient refusal to participate in the study.

### Study intervention

This study was based on data derived from MPMRs implemented on January 1st 2020.

MPMRs were introduced to regularly reassess the benefit–risk balance of each medication prescribed to patients in the psychiatric wards where the reviews were conducted. The primary objective of these meetings was to promote the safe and appropriate use of medicines.

MPMRs took place in four psychiatric wards of the hospital, held weekly, and lasted approximately 1.5 to 2 hours each. They were conducted collaboratively by a multidisciplinary team comprising clinical hospital pharmacist(s), psychiatrist(s), general practitioner(s), nurses, and other staff members involved in patient care.

Each review began with a nurse’s summary of the patient’s current clinical status and, when possible, a report of the patient’s own perspective. The patient’s medication regimen was then discussed collectively, taking into account observed symptomatology, somatic comorbidities, and the management of adverse effects. Based on this discussion, the treatment plan was updated collaboratively, and therapeutic objectives for the following weeks were defined.

Any pharmacological questions raised during the meeting were noted by the pharmacist, who subsequently provided a documented, evidence-based response by email within the week, accompanied by an oral explanation during the next MPMR if necessary.

These MPMRs constituted the intervention in the present study.

### Outcome variables

In this study, the primary outcome was the change in the number of prescribed medications associated with the implementation of MPMRs. It was calculated as the difference in the number of medications prescribed at admission and at discharge, and compared pre- and post-MPMR implementation. Considering both initial and final prescriptions allowed us to account for treatment adjustments made during hospitalization.

This primary outcome was selected given the well-established association between an increasing number of prescribed medications and the occurrence of diverse DRPs, as highlighted in a 2023 systematic review including 11 studies [30]. We hypothesized that MPMRs, through their shared decision-making approach, would promote evidence-based prescribing and ultimately reduce polypharmacy.

Secondary outcomes focused on changes in prescriptions for psychotropic medications overall (i), as well as for selected psychotropic subclasses analyzed separately (benzodiazepines (ii), antipsychotics (iii), antidepressants (iv), and mood stabilizers (v)). We also assessed anticholinergic agents (vi), laxatives (vii), and analgesics (viii) because of their relevance to the management of psychotropic treatment and its adverse effects [31,32].

The number of prescribed benzodiazepines (ii) was used as a proxy indicator of compliance with French recommendations, given that benzodiazepine use is considered excessive in France [33,34].

The Anatomical Therapeutic Chemical (ATC) Classification System was used to identify medications belonging to each category. The ATC codes used to define each medication category and subclass are provided in S1 Table.

### Data collection

Data for this study were extracted retrospectively from the electronic medical records of the Centre Hospitalier Le Vinatier (Bron, France) on 29/08/2024. All medication prescriptions, including dates of initiation, modification, and discontinuation, were documented electronically. Basic sociodemographic information (age and sex) was also collected. Additional clinical data, such as admission details and diagnoses coded according to the International Classification of Diseases, 10th Revision (ICD-10, World Health Organization), were obtained from the same records.

Data collection was conducted using anonymized datasets to ensure participant confidentiality. Identifiable personal information (e.g., name, address, contact details) was not recorded. Each participant was assigned a unique identification code for analysis, and only fully anonymized data were used for statistical processing. Data were stored in a secure system accessible exclusively to authorized research personnel.

The Anatomical Therapeutic Chemical (ATC) Classification System was used to classify medications and assess secondary outcomes (S1 Table).

### Consent to participate declaration and ethical approval

This retrospective study used data collected during routine clinical care. In accordance with French regulations, the requirement for written informed consent was waived by the Ethics Committee due to the retrospective design and use of anonymized data. Patients were informed of the use of their data through an information and non-opposition notice delivered in person, by mail, or by email according to usual communication methods, and via a notice published on the institutional website; data were included unless patients explicitly objected. The study was approved by the Ethics Committee of Centre Hospitalier Le Vinatier (CEREVI/2024/023; June 6^th^, 2024) and conducted in accordance with the Declaration of Helsinki.

### Data pre-processing

The dataset was examined for completeness, and no missing values were identified. In psychiatric settings, the length of hospital stay varies considerably, ranging from a few days to several weeks or months. Extreme values in duration of stay can influence analyses when assessing the impact of MPMRs. Longer stays may reflect greater clinical complexity or organizational factors, whereas very short stays may be unrelated to the intervention, potentially introducing bias.

To reduce this variability, we applied an inclusion threshold of a minimum two-week hospital stay, ensuring that all included patients had the opportunity to receive at least one MPMR. Following this selection, we assessed the distribution of the data and screened for outliers. The 1.5 interquartile range (IQR) rule was applied to determine upper bounds for both cohorts [35].

### Data analysis

All analyses were performed using Python 3.8.8 in a Jupyter Notebook environment.

#### Descriptive statistics.

Baseline characteristics were summarized using descriptive statistics: mean and standard deviation (SD) or median and interquartile range (IQR) for continuous variables, and frequencies and percentages for categorical variables. To assess normality, inspection of Q-Q plots and Shapiro-Wilk tests were used.

#### Comparative analyses.

For each outcome, the change between admission and discharge was calculated. For the primary outcome (change in total medication count from admission to discharge, pre vs. post MPMR implementation), comparisons between the pre- and post-MPMR periods were performed using paired t-tests for normally distributed variables or the Wilcoxon signed-rank test for non-normally distributed variables. Secondary outcomes, assessing changes within specific drug classes, were analyzed using the same approach. Effect sizes were reported as Cohen’s *d* for normally distributed data and as Pearson’s r for non-parametric tests (r = Z/√N). A significance level of α = 0.05 was applied.

To strengthen the analysis, a Mixed Linear Model (MLM) was applied with patient ID as a random effect to account for the repeated-measures structure (two time points per patient: pre and post MPMR implementation). Fixed effects included age, sex, duration of hospitalization, number of MPMRs received, and ward. Because previous studies have suggested that sex may influence the clinical response to psychotropic medications and treatment outcomes in patients receiving antipsychotic therapy, sex was explored as a potential effect modifier by including an interaction term with the intervention period in the mixed linear model [36–39]. This model allowed for adjustment of individual-level variability and improved the accuracy of the estimated effect of MPMR on prescribing outcomes. The MLM was applied to the primary outcome and to secondary outcome measures concerning the overall psychotropic medications only, as the remaining drug subclasses did not have sufficient data to support reliable model estimation.

## Results

### Participants and descriptive data

The study retrospectively included 120 patients who had two hospital stays and received at least one MPMR during their second stay (after MPMR implementation stay). There were no missing values.

Table 1 presents patient characteristics.

**Table 1:** Patient characteristics.

Variables	Before MPMR	After 1 + MPMR
Age (years), median (IQR)	44 (19)	45.5 (20.3)
Sex (%)	M: 71 (59.2%) F: 49 (40.8%)	
Hospital stay duration, median (IQR)	42 (42.5)	36 (30.3)
Main diagnosis, ICD-10 code (%)	F2: Schizophrenia or related, 56 (46.7%) F3: Mood disorders, 17 (14.2%) F4: Neurotic, stress-related and somatoform disorders, 11 (9.2%) F6: Personality and behaviour disorders, 11 (9.2%) F1: Substance-related disorders, 9 (7.5%) Other diagnosis, 16 (13.3%)	

**Note.** N = 120. MPMR: Multi-professional medication review. IQR: interquartile range. M: male. F: female. Psychiatric diagnoses are based on the International Classification of Diseases (ICD), 10^th^ Revision (World Health Organization), with codes beginning with “F”.

Other descriptive results can be found in S2 Table, which provides data on the number of molecules per class in hospital discharge prescriptions, pre- and post-MPMR implementation.

There was no statistical difference in the median hospital stay duration pre- and post-MPMR implementation.

### Primary outcome

Results regarding the primary outcome can be found in Table 2.

**Table 2:** Impacts on medication prescriptions of one or more multi-professional medication reviews (MPMR), n = 120.

Outcomes	Before MPMR	After 1 or + MPMR^i^	Mean Δ (sd)	p-value ^ii^	Effect Size ^iii^	p-value (MLM*)
Average change in the number of **medications** prescribed per patient, mean (sd)	+0.4 (3.8)	−0.7 (4.9)	−1.1 (6.0)	0.045	0.18	0.001
Average change in the number of **psychotropic drugs** prescribed per patient, median (IQR)	0 (3)	−1 (4)	−1.5 (3.5)	<0.001	0.43	<0.001
Average change in the number of **benzodiazepines** prescribed per patient, median (IQR)	0 (1)	−1 (2)	−0.8 (1.9)	<0.001	0.40	NA
Average change in the number of **antipsychotics** prescribed per patient, median (IQR)	0 (2)	0 (3)	−0.3 (2.0)	0.009	0.34	NA
Average change in the number of **antidepressants** prescribed per patient, median (IQR)	0 (0)	0 (0)	0.1 (0.3)	0.278	NA	NA
Average change in the number of **mood stabilizers** prescribed per patient, median (IQR)	0 (0)	0 (0)	−0.5 (0.8)	0.218	NA	NA
Average change in the number of **anticholinergic agents** prescribed per patient, median (IQR)	0 (0)	0 (0)	−0.7 (0.9)	0.009	0.36	NA
Average change in the number of **laxatives** prescribed per patient, median (IQR)	0 (0.3)	0 (1)	−0.2 (1.2)	0.013	0.34	NA
Average change in the number of **analgesic drugs** prescribed per patient, median (IQR)	0 (0)	0 (0)	0.3 (0.6)	0.082	NA	NA

**Note.** Average changes are calculated during hospital stays, before and after the intervention.

MPMR: Multi-professional medication review; sd: Standard deviation; IQR: Interquartile range

I The last known medication prescription was considered for the analyses after a minimum of one MPMR, during the first hospital stay in which the patient received the intervention.

Ii P-value calculated with paired t-test or Wilcoxon signed-rank test.

Iii Effect size calculated with Cohen’s *d* or (r). For normally distributed values, Cohen’s d was used, and its value can be interpreted as follows: **0.2**: Small effect size, **0.5**: Medium effect size and **0.8**: Large effect size. For non-parametric Wilcoxon signed-rank test, the effect size was reported as **r**, which is derived from the z-value provided by the test and the sample size. (**0.1**: Small effect size; **0.3**: Medium effect size; **0.5**: Large effect size).

* Mixed Linear Model.

Among participants, the average change in the number of prescribed medications showed a significant decrease following one or more multi-professional medication reviews (MPMR). Prior to the implementation of MPMR, there was a slight increase in the number of medications prescribed between admission and discharge, with a mean increase of +0.4 (SD = 3.8). After the introduction of MPMR, this trend reversed, with a decrease of −0.7 (SD = 4.9). The overall mean differential between pre- and post-intervention prescriptions was −1.1 (SD = 6.0), with a p-value of 0.045. However, the practical significance of this difference is considered small, as indicated by Cohen’s *d* of 0.18.

The mixed linear model confirms this observation, with Epoque (the intervention period: pre or post MPMR) significantly associated with a reduced number of prescribed medications (β = −0.658, p = 0.001). Other factors, including gender, age, length of stay, and number of MPMR received did not show significant impacts on the differential of medication prescriptions. Furthermore, no statistical difference between the units were observed with the MLM in terms of differential of medication prescriptions.

A detailed analysis of the residuals showed an overall normal distribution and homogeneous variance, attesting the validity of our results. The detailed results of the MLM output can be found in S3 Table.

### Secondary outcomes

#### Overall psychotropic medications.

The number of prescribed psychotropic medications (overall) significantly decreased following the implementation of MPMR, with a mean change of −1.5 (SD = 3.5; p = 0.001). The effect size was small to moderate (Cohen’s *d* = −0.43). This reduction was further supported by the MLM, which confirmed a significant effect of the intervention, sex, and their interaction on the change in psychotropic prescriptions: Intervention (β = −2.04, p < 0.001), Sex (reference: M = 0) (β = −1.18, p = 0.012), and Intervention × Sex (β = 1.76, p < 0.001). Model diagnostics indicated normally distributed residuals and homogeneous variance, supporting the validity of the results.

#### Benzodiazepines.

The number of prescribed benzodiazepines significantly decreased following the implementation of MPMR. Before the implementation of MPMR, the median differential for benzodiazepines was 0 (IQR = 1), whereas after MPMR, the differential decreased to −1 (IQR = 2). The overall mean differential was −0.8 (SD = 1.9), with a p-value < 0.001, indicating statistical significance, and an effect size of Cohen’s *d* = −0.40, suggesting a small to moderate effect.

#### Antipsychotics.

The number of prescribed antipsychotics significantly decreased following the implementation of MPMR. The overall mean differential between pre/post MPMR was −0.3 (SD = 2.0), reaching statistical significance (p = 0.009, Cohen’s *d* = −0.34).

#### Antidepressants.

Regarding antidepressants, no statistically significant results were found following the implementation of MPMR.

#### Mood stabilizers.

Regarding mood stabilizers, no statistically significant results were found following the implementation of MPMR.

#### Other somatic drugs.

Statistically significant changes were observed for both anticholinergic agents and laxatives following the implementation of MPMR. The mean change in anticholinergic agent use was −0.7 (SD = 0.9; p = 0.009), and −0.2 (SD = 1.2; p = 0.013) for laxatives. In contrast, no significant change was observed in analgesic drug use before and after the introduction of MPMR.

## Discussion

### Key findings

This study retrospectively evaluated the impact of hospital-based multi-professional medication reviews (MPMRs) in a psychiatric setting and demonstrated that MPMRs were associated with a reduction in the number of medications prescribed at discharge for 120 patients (−1.1 drugs prescribed, SD = 6.0, p = 0.045 or 0.001 with a MLM, *d* = 0.18).

Although the overall effect size was small, reductions particularly concerned drug classes known to increase the risk of adverse effects and inappropriate prescribing in psychiatric care [40,41]. Notably, prescriptions of overall psychotropics, especially benzodiazepines, and anticholinergic agents declined following MPMR implementation, while no significant changes were observed for antidepressants, mood stabilizers, or analgesics.

### Interpretation and comparison with existing literature

Our findings are consistent with evidence indicating that medication reviews can reduce polypharmacy and improve prescribing quality. A 2017 systematic review and meta-analysis of randomized controlled trials on the effectiveness of medication review found that 12 studies with low risk of bias indicated a greater decrease or smaller increase of the number of drugs used [42]. Similarly, despite the small change in total medication count observed in our study, the targeted reduction of potentially inappropriate or high-risk medications suggests clinically meaningful benefits.

Psychotropic medications, particularly benzodiazepines, represented a key area of improvement. Their reduction aligns with international deprescribing recommendations and with previous meta-analyses showing that structured medication review interventions can effectively reduce benzodiazepine use [33,34,43–45].

In our sample, the proportion of patients discharged without benzodiazepines increased markedly following MPMR implementation, reflecting a shift toward safer prescribing practices.

We also observed a small decrease in antipsychotic prescriptions. Given that antipsychotics represent essential treatment for many patients in our cohort, particularly those with schizophrenia spectrum disorders, large reductions would not be expected. Nonetheless, even a modest decline may reflect an effort to reduce unnecessary polypharmacy and promote antipsychotic monotherapy, an approach consistently recommended to minimize side effects and drug interactions [40].

Sex differences emerged in the change in psychotropic prescriptions, with men showing a greater reduction than women. Literature has identified sex-specific patterns in psychotropic response and prescribing practices, which may partly explain this differential effect, although further investigation is warranted [36–39].

Beyond psychotropics, a substantial reduction in anticholinergic agents was observed. These medications, although useful in some cases to manage antipsychotics’ side effects such as dyskinesia or sialorrhea, are associated with cognitive impairment and other adverse effects and are recommended for restrictive use [46,47]. Their decrease after MPMR suggests enhanced medication appropriateness and attention to somatic safety within psychiatric prescribing.

### Clinical implications

Although the reduction in total medications was modest, the focus on deprescribing high-risk and potentially inappropriate medications suggests a meaningful contribution to patient safety and quality of care. By systematically addressing medication-related problems during hospitalization, MPMRs may help prevent adverse effects, improve treatment acceptability, and contribute to better long-term outcomes, including potentially reducing avoidable readmissions. These findings support the value of implementing structured MPMRs in psychiatric inpatient settings, where complex polypharmacy remains prevalent.

### Strengths and limitations

A key strength of this study is its within-patient pre-post design, which limits inter-individual variability and enhances internal validity. The systematic and routine implementation of MPMRs for all inpatients further increases the reliability of the intervention’s observed effects.

However, several limitations must be acknowledged. First, the non-randomized pre–post design cannot fully rule out confounding factors or temporal changes in patients’ clinical status. Because psychiatric disorders often have fluctuating courses and treatment requirements evolve over time, some observed changes in prescribing may reflect changes in disease severity or clinical presentation rather than the MPMR intervention itself. A randomized controlled trial would provide stronger evidence of causality. Second, requiring two admissions for inclusion may have introduced selection bias, potentially favoring more severe or complex patients. Third, the study focused primarily on quantitative prescription changes, limiting the ability to capture qualitative improvements, such as dose optimization, deprescribing of pro re nata medications, or enhanced monitoring strategies.

### Perspectives

Future research should prioritize controlled study designs, ideally randomized trials, to more robustly assess the effectiveness of MPMRs in psychiatry. In addition, integrating key components identified in existing realist reviews, such as clear role definitions within the multidisciplinary team, structured processes, interprofessional communication, and active involvement of patients and carers, may strengthen MPMR implementation and enhance its impact in psychiatric settings [48]. Exploring patient-reported outcomes, functional recovery, and long-term effects on readmission rates will also be critical to understanding the broader value of MPMRs.

## Conclusion

In summary, this study suggests that the implementation of MPMRs in a psychiatric inpatient setting may improve prescribing practices, particularly through the reduction of high-risk medications. While the overall quantitative impact was modest (−1.1 drugs prescribed, SD = 6.0, p = 0.045 or 0.001 with a MLM, *d* = 0.18), the findings highlight the potential of structured, multidisciplinary reviews to enhance medication appropriateness and patient safety. Continued refinement of MPMR models and evaluation through robust study designs are warranted to confirm and expand on these results.

## Supporting information

S1 Table List of anatomical therapeutic chemical (ATC) classification system codes used. (DOCX)

S2 Table Number of molecules per class in hospital discharge prescriptions, before and after MPMR implementation. (DOCX)

S3 Table Mixed linear model (MLM) regression output regarding primary outcome. (DOCX)
