# Supplementary material for: Impact of multi-professional medication reviews (MPMRs) on medication prescriptions in psychiatry
Source: PLoS One. 2026 Sep 21;21(9):e0358539. doi: 10.1371/journal.pone.0358539 (PMC13592593; doi:10.1371/journal.pone.0358539)

Jen Edwards
Staff Editor, *Plos One*
Revised version submission date: June 22nd, 2026
ID of initial submission: PONE-D-26-11858

Matthieu LEBRAT
PharmD, MPH
Hospices Civils de Lyon, Lyon, France
matthieu.lebrat@chu-lyon.fr
Tel: +33 478863353

Manuscript title: Impact of Multi-Professional Medication Reviews (MPMRs) on medication prescriptions in psychiatry

Dear Jen Edwards,

We would like to thank you and reviewer 1 for the feedback provided on our manuscript. We sincerely appreciate the time and effort invested in reviewing our work, as well as the valuable suggestions offered to improve it.

In response to the Editor's and reviewer's suggestions, we have revised the manuscript accordingly and are pleased to submit this revision. Below, we provide a detailed, point-by-point response to each comment. For ease of reference, the reviewers' comments are reproduced in **bold**, followed by our responses. All revisions made to the manuscript are indicated with line numbers.

Point by point response to Editor:

Regarding the comment 1: "**Please ensure that your manuscript meets PLOS ONE's style requirements, including those for file naming.**"

Author's response: We thank the Editor for this comment. A careful review of the manuscript has been conducted, and it has been updated to meet PLOS ONE's style requirements.

Regarding the comment 2: "**In the online submission form you indicate that your data is not available for proprietary reasons and have provided a contact point for accessing this data. Please note that your current contact point is a co-author on this manuscript. According to our Data Policy, the contact point must not be an author on the manuscript and must be an institutional contact, ideally not an individual. Please revise your data statement to a non-author institutional point of contact, such as a data access or ethics committee, and send this to us via return email. Please also include contact information for the third party organization, and please include the full citation of where the data can be found.**"

Author's response: We thank the Editor for this comment. We have revised the Data Availability Statement to provide a non-author institutional contact, in accordance with the journal's Data Policy. The institutional contact for the Ethics Committee of Centre Hospitalier Le Vinatier has been provided as the point of contact for data access (e-mail: CEREVI@ch-le-vinatier.fr). The full citation describing the data source is included in the manuscript, under the "Data availability" section.

Point by point response to Reviewer 1:

Regarding the comment 1: "**The paper is essentially describing the role of the MDT in delivery of pharmaceutical care, specifically assessing appropriateness of medication at discharge. The authors state that there is little known about this within psychiatry/in-patient care (lines 126-134) as the literature tends to focus on the role of pharmacy/pharmacists to conduct med reviews etc. Nevertheless, the topic has moved on considerably from the state that is suggested by the authors in the introduction. More recent and relevant references could be used (avoid anything >20yrs old)"**

Author's response: We thank the reviewer for this relevant suggestion. We have updated the Introduction to incorporate recent literature and better reflect the current state of evidence regarding multidisciplinary medication management in psychiatric settings.

At line 117, we updated the text as follows:

"Increasing evidence supports the integration of clinical pharmacists within multidisciplinary mental health teams, where their involvement improves the identification of drug-related problems, medication reconciliation, prescribing quality, and medication safety [19, and new Ref. DOI: 10.1007/s11096-022-01409-4]"

We also tempered our wording by replacing "largely unexplored" with "less explored in the literature", to better reflect the current evidence base.

To acknowledge recent developments in the field, we added the following paragraph with new references (line 147):

"Recent evidence has expanded the literature on multidisciplinary medication management in psychiatric settings. Several studies have demonstrated that structured interdisciplinary approaches involving pharmacists improve medication safety during transitions of care and reduce medication-related problems. In a quasi-experimental study conducted in a psychogeriatric unit, interdisciplinary medication reconciliation at discharge significantly reduced reconciliation errors from 55.2% to 37% (DOI: 10.1016/j.gaceta.2025.102501). Likewise, a quality-improvement project in an inpatient psychiatry unit identified numerous prescribing errors, highlighting the value of systematic multidisciplinary review (DOI:10.9740/mhc.2024.04.097), while a pilot study in a psychiatric emergency department demonstrated significant reductions in medication discrepancies following pharmacist-supported medication reconciliation (DOI: 10.1007/s40267-021-00824-3)."

Accordingly, we have reframed the rationale for the study to acknowledge the growing body of evidence supporting multidisciplinary medication management in psychiatric settings, while clarifying that our study addresses the comparatively underexplored model of routine, ward-wide multidisciplinary medication review.

Line 164, we modified: "Given the complexity of psychiatric pharmacotherapy, effective medication management for individuals with mental disorders requires a highly specialized and collaborative approach that MPMR could bring. Evidence documenting its impact in psychiatry remains scarce."

By:

"Nevertheless, these interventions mainly focus on medication reconciliation at transitions of care, discharge optimisation, or targeted pharmacist-led reviews. While multidisciplinary medication optimisation in psychiatric settings has been increasingly described in recent years, this ward-wide multidisciplinary medication review model, in which all prescriptions for all psychiatric inpatients are jointly reassessed during routine clinical practice, has received comparatively little attention. This distinction underpins the rationale for the present study."

Regarding the comment 2: "**The methodology is appropriate- perhaps call it a pre-post study and not a before and after (line 151).**"

Author's response: We thank the reviewer for this helpful suggestion. We have adopted the term "pre-post study" throughout the manuscript to ensure consistent terminology.

Regarding the comment 3: "**Line 160 should say MPMRs were implemented on 1/1/2020 instead of have been.**"

Author's response: We thank the reviewer for this helpful suggestion. We have revised the manuscript accordingly. The sentence now reads (line 188):

"MPMRs were implemented on 1/1/2020."

Regarding the comment 4: "**line 174 states that the design restricts bias but it should be acknowledged that most mental illness have variable presentation and treatment types and doses will vary throughout the lifetime so the prescribing patterns may reflect he patients current presentation and not necessarily all be due to the intervention.**"

Author's response: We thank the reviewer for this insightful comment. We agree that, although using each patient as their own comparator reduces inter-individual variability, changes in prescribing over time may also reflect the fluctuating course of psychiatric disorders and evolving treatment needs rather than the intervention alone. We have therefore revised the Methods section (line 196) to clarify this point and have also strengthened the Limitations section to explicitly acknowledge the potential influence of changes in patients' clinical presentation over time:

The Method section now reads (line 196):

"The use of each patient's pre-intervention stay as a comparator aimed to minimize potential confounding factors, such as inter-individual variability and differences in prescribing practices between patients. However, given the fluctuating nature of psychiatric disorders and the evolution of patients' clinical status over time, changes in prescribing patterns may also reflect variations in disease presentation, treatment response, or illness progression, rather than the effect of the intervention alone."

The Limitation section now reads (line 481):

"First, the non-randomized pre–post design cannot fully rule out confounding factors or temporal changes in patients' clinical status. Because psychiatric disorders often have fluctuating courses and treatment requirements evolve over time, some observed changes in prescribing may reflect changes in disease severity or clinical presentation rather than the MPMR intervention itself."

Regarding the comment 5: "**More details are needed about the issue of 'sex related differences in response to psychotropic medication' (line 289)- The references suggest variance in outcomes (which is more complicated than response to medication) and/or dose response/dosages used. However, most antipsychotic meds are not prescribed according to weight (mg/kg) and therefore not impacted by sex. This statement therefore requires more detail/clarification."**

Author's response: We thank the reviewer for this thoughtful comment. We agree that our original wording lacked precision and could be interpreted as implying that psychotropic medications are routinely prescribed according to sex or body weight. Our intention was instead to justify the inclusion of sex as a potential effect modifier in the mixed linear model, based on previous literature suggesting sex-related differences in treatment response and clinical outcomes associated with psychotropic medications. We have therefore revised the Methods section to clarify this rationale and have also replaced one of the 3 previous references with 2 more appropriate references specifically addressing sex differences in psychopharmacology (line 317):

"Because previous studies have suggested that sex may influence the clinical response to psychotropic medications and treatment outcomes in patients receiving antipsychotic therapy, sex was explored as a potential effect modifier by including an interaction term with the intervention period in the mixed linear model [32–34] (And we replaced Ref. 32 by DOI: 10.1002/wps.21155, and DOI: 10.3390/jpm16040189)"

Regarding the comment 6: "**The use of the term psychotropic medication as a category is misleading, esp as it is given as a separate entity from Benzo's, antipsychotopics and anticholinergics which are themselves also psychotropic meds. I suspect this category contains antidepressants/mood stabilizers and other CNS medication but this detail should be provided in the results/methods."**

Author's response: We thank the reviewer for this helpful comment. We agree that the presentation of the medication categories could have been clearer. Our intention was for "psychotropic medications" to represent the overall category, while benzodiazepines, antipsychotics, antidepressants, and mood stabilizers were analysed separately as psychotropic subclasses. Anticholinergic agents, laxatives, and analgesics were included as additional medication categories because of their relevance to the management of psychotropic treatment and its adverse effects. To avoid ambiguity, we have revised the Methods section to clarify this categorization and now explicitly state that the corresponding ATC codes defining each category and subclass are provided in S1 Table. We have also amended the Results section by referring to overall psychotropic medications, as well as throughout the manuscript, to distinguish this analysis from those of the individual subclasses.

Line 246, we modified:

"Secondary outcomes focused on specific drug categories to explore more targeted prescribing changes. We assessed the impact of MPMRs on the change in the number of prescriptions for the following drug classes: (i) psychotropic drugs, (ii) benzodiazepines, (iii) antipsychotics, (iv) antidepressants, (v) mood stabilizers, (vi) anticholinergic agents, (vii) laxatives and (viii) analgesic drugs.
Anticholinergic agents (vi) and laxatives (vii) were examined to evaluate the impact of MPMRs on the management of common adverse events in psychiatric settings, as these two classes are frequently used to manage psychotropic-induced side effects [27,28].
The number of prescribed benzodiazepines (ii) was used as a proxy indicator of compliance with French recommendations, given that benzodiazepine use is considered excessive in France [29,30].
The Anatomical Therapeutic Chemical (ATC) Classification System was used to identify medications belonging to each category. The full list of ATC codes used in this study is available in Supplementary File I."

By:

"Secondary outcomes focused on changes in prescriptions for psychotropic medications overall (i), as well as for selected psychotropic subclasses analysed separately (benzodiazepines (ii), antipsychotics (iii), antidepressants (iv), and mood stabilizers (v)). We also assessed anticholinergic agents (vi), laxatives (vii), and analgesics (viii) because of their relevance to the management of psychotropic treatment and its adverse effects [27,28].
The number of prescribed benzodiazepines (ii) was used as a proxy indicator of compliance with French recommendations, given that benzodiazepine use is considered excessive in France [29,30].
The Anatomical Therapeutic Chemical (ATC) Classification System was used to identify medications belonging to each category. The ATC codes used to define each medication category and subclass are provided in S1 Table."

In the result section, under the "secondary outcomes", we have modified the title as follows line 382:

"Overall psychotropic medications"

Regarding the comment 7: "**Numerical values should be reported with appropriate decimal places (one decimal place is often sufficient e.g SD= 20.2, 3.8) and a ZERO should also be inserted prior to the decimal point e.g 0.5, 0.05 etc."**

<u>Author's response</u>: We thank the reviewer for this helpful suggestion. We have revised the numerical formatting throughout the manuscript to improve consistency and readability. Descriptive statistics are now reported using an appropriate number of decimal places (generally one decimal place), while inferential statistics (e.g., regression coefficients and p-values) are reported according to standard statistical reporting conventions. In addition, leading zeros have been added before decimal points throughout the manuscript.

We hope that these revisions adequately address all concerns and that the revised manuscript will be suitable for publication in Plos One.

Sincerely,

Matthieu Lebrat, corresponding author.

On behalf of all authors.

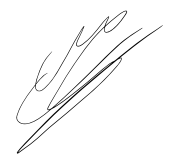

Supplement: Supplementary file 1 [file pone.0358539.s005.pdf]
