# Supplementary material for: Impact of multi-professional medication reviews (MPMRs) on medication prescriptions in psychiatry
Source: PLoS One. 2026 Sep 21;21(9):e0358539. doi: 10.1371/journal.pone.0358539 (PMC13592593; doi:10.1371/journal.pone.0358539)
Supplement: Supplementary file 2 [file pone.0358539.s001.docx]

**S1 Table**. **List of Anatomical Therapeutic Chemical (ATC) Classification System codes used.**

| Psychotropic drugs | | | | | | |
| --- | --- | --- | --- | --- | --- | --- |
| N06AA01 | N06AX01 | N05AB07 | N05AH08 | N05AH46 | N05BA13 | N05BA51 |
| N06AA02 | N06AX02 | N05AB08 | N05AH09 | N05AH47 | N05BA14 | N05BA52 |
| N06AA03 | N06AX03 | N05AB09 | N05AH10 | N05AH48 | N05BA15 | N05BA53 |
| N06AA04 | N06AX04 | N05AB10 | N05AH11 | N05AH49 | N05BA16 | N05BA54 |
| N06AA05 | N06AX05 | N05AC01 | N05AH12 | N05AH50 | N05BA17 | N05BA55 |
| N06AA06 | N06AX06 | N05AC02 | N05AH13 | N05AH51 | N05BA18 | N05BA56 |
| N06AA07 | N06AX07 | N05AC03 | N05AH14 | N05AH52 | N05BA19 | N03AE01 |
| N06AA08 | N06AX08 | N05AC04 | N05AH15 | N05AH53 | N05BA20 | N05CD01 |
| N06AA09 | N06AX09 | N05AD01 | N05AH16 | N05AL01 | N05BA21 | N05CD02 |
| N06AA10 | N06AX10 | N05AD02 | N05AH17 | N05AL02 | N05BA22 | N05CD03 |
| N06AA11 | N06AX11 | N05AD03 | N05AH18 | N05AL03 | N05BA23 | N05CD04 |
| N06AA12 | N06AX12 | N05AD04 | N05AH19 | N05AL04 | N05BA24 | N05CD05 |
| N06AA13 | N06AX13 | N05AD05 | N05AH20 | N05AL05 | N05BA25 | N05CD06 |
| N06AA14 | N06AX14 | N05AD06 | N05AH21 | N05AL06 | N05BA26 | N05CD07 |
| N06AA15 | N06AX15 | N05AD07 | N05AH22 | N05AL07 | N05BA27 | N05CD08 |
| N06AA16 | N06AX16 | N05AD08 | N05AH23 | N05AX07 | N05BA28 | N05CD09 |
| N06AA17 | N06AX17 | N05AD09 | N05AH24 | N05AX08 | N05BA29 | N05CD10 |
| N06AA18 | N06AX18 | N05AD10 | N05AH25 | N05AX09 | N05BA30 | N05CD11 |
| N06AA19 | N06AX19 | N05AE01 | N05AH26 | N05AX10 | N05BA31 | N05CD12 |
| N06AA21 | N06AX21 | N05AE02 | N05AH27 | N05AX11 | N05BA32 | N05CD13 |
| N06AA23 | N06AX22 | N05AE03 | N05AH28 | N05AX12 | N05BA33 | N05CD14 |
| N06AB02 | N06AX23 | N05AE04 | N05AH29 | N05AX13 | N05BA34 | N05CD15 |
| N06AB03 | N06AX24 | N05AE05 | N05AH30 | N05AX14 | N05BA35 | N05CF01 |
| N06AB04 | N06AX25 | N05AF01 | N05AH31 | N05AX15 | N05BA36 | N05CF02 |
| N06AB05 | N06AX26 | N05AF02 | N05AH32 | N05AX16 | N05BA37 | N05CF03 |
| N06AB06 | N05AA01 | N05AF03 | N05AH33 | N05AX17 | N05BA38 | N05CF04 |
| N06AB07 | N05AA02 | N05AF04 | N05AH34 | N05BA01 | N05BA39 | N03AE01 |
| N06AB08 | N05AA03 | N05AF05 | N05AH35 | N05BA02 | N05BA40 | N05AN01 |
| N06AB09 | N05AA04 | N05AG01 | N05AH36 | N05BA03 | N05BA41 | N03AG01 |
| N06AB10 | N05AA05 | N05AG02 | N05AH37 | N05BA04 | N05BA42 | N03AG02 |
| N06AF01 | N05AA06 | N05AG03 | N05AH38 | N05BA05 | N05BA43 | N03AG03 |
| N06AF02 | N05AA07 | N05AH01 | N05AH39 | N05BA06 | N05BA44 | N03AG04 |
| N06AF03 | N05AB01 | N05AH02 | N05AH40 | N05BA07 | N05BA45 | N03AG05 |
| N06AF04 | N05AB02 | N05AH03 | N05AH41 | N05BA08 | N05BA46 | N03AG06 |
| N06AF05 | N05AB03 | N05AH04 | N05AH42 | N05BA09 | N05BA47 | N03AX09 |
| N06AF06 | N05AB04 | N05AH05 | N05AH43 | N05BA10 | N05BA48 | NO3AF01 |
| N06AG02 | N05AB05 | N05AH06 | N05AH44 | N05BA11 | N05BA49 |  |
| N06AG03 | N05AB06 | N05AH07 | N05AH45 | N05BA12 | N05BA50 |  |

| Antipsychotics | | |  | Antidepressants | |
| --- | --- | --- | --- | --- | --- |
| N05AA01 | N05AF03 | N05AH33 |  | N06AA01 | N06AX01 |
| N05AA02 | N05AF04 | N05AH34 |  | N06AA02 | N06AX02 |
| N05AA03 | N05AF05 | N05AH35 |  | N06AA03 | N06AX03 |
| N05AA04 | N05AG01 | N05AH36 |  | N06AA04 | N06AX04 |
| N05AA05 | N05AG02 | N05AH37 |  | N06AA05 | N06AX05 |
| N05AA06 | N05AG03 | N05AH38 |  | N06AA06 | N06AX06 |
| N05AA07 | N05AH01 | N05AH39 |  | N06AA07 | N06AX07 |
| N05AB01 | N05AH02 | N05AH40 |  | N06AA08 | N06AX08 |
| N05AB02 | N05AH03 | N05AH41 |  | N06AA09 | N06AX09 |
| N05AB03 | N05AH04 | N05AH42 |  | N06AA10 | N06AX10 |
| N05AB04 | N05AH05 | N05AH43 |  | N06AA11 | N06AX11 |
| N05AB05 | N05AH06 | N05AH44 |  | N06AA12 | N06AX12 |
| N05AB06 | N05AH07 | N05AH45 |  | N06AA13 | N06AX13 |
| N05AB07 | N05AH08 | N05AH46 |  | N06AA14 | N06AX14 |
| N05AB08 | N05AH09 | N05AH47 |  | N06AA15 | N06AX15 |
| N05AB09 | N05AH10 | N05AH48 |  | N06AA16 | N06AX16 |
| N05AB10 | N05AH11 | N05AH49 |  | N06AA17 | N06AX17 |
| N05AC01 | N05AH12 | N05AH50 |  | N06AA18 | N06AX18 |
| N05AC02 | N05AH13 | N05AH51 |  | N06AA19 | N06AX19 |
| N05AC03 | N05AH14 | N05AH52 |  | N06AA21 | N06AX21 |
| N05AC04 | N05AH15 | N05AH53 |  | N06AA23 | N06AX22 |
| N05AD01 | N05AH16 | N05AL01 |  | N06AB02 | N06AX23 |
| N05AD02 | N05AH17 | N05AL02 |  | N06AB03 | N06AX24 |
| N05AD03 | N05AH18 | N05AL03 |  | N06AB04 | N06AX25 |
| N05AD04 | N05AH19 | N05AL04 |  | N06AB05 | N06AX26 |
| N05AD05 | N05AH20 | N05AL05 |  | N06AB06 |  |
| N05AD06 | N05AH21 | N05AL06 |  | N06AB07 |  |
| N05AD07 | N05AH22 | N05AL07 |  | N06AB08 |  |
| N05AD08 | N05AH23 | N05AX07 |  | N06AB09 |  |
| N05AD09 | N05AH24 | N05AX08 |  | N06AB10 |  |
| N05AD10 | N05AH25 | N05AX09 |  | N06AF01 |  |
| N05AE01 | N05AH26 | N05AX10 |  | N06AF02 |  |
| N05AE02 | N05AH27 | N05AX11 |  | N06AF03 |  |
| N05AE03 | N05AH28 | N05AX12 |  | N06AF04 |  |
| N05AE04 | N05AH29 | N05AX13 |  | N06AF05 |  |
| N05AE05 | N05AH30 | N05AX14 |  | N06AF06 |  |
| N05AF01 | N05AH31 | N05AX15 |  | N06AG02 |  |
| N05AF02 | N05AH32 | N05AX16 |  | N06AG03 |  |
|  |  | N05AX17 |  |  |  |

| Mood Stabilizers |  | Benzodiazepines | |
| --- | --- | --- | --- |
| N05AN01 |  | N05BA01 | N05BA39 |
| N03AG01 |  | N05BA02 | N05BA40 |
| N03AG02 |  | N05BA03 | N05BA41 |
| N03AG03 |  | N05BA04 | N05BA42 |
| N03AG04 |  | N05BA05 | N05BA43 |
| N03AG05 |  | N05BA06 | N05BA44 |
| N03AG06 |  | N05BA07 | N05BA45 |
| N03AX09 |  | N05BA08 | N05BA46 |
| N03AF01 |  | N05BA09 | N05BA47 |
| N03AF02 |  | N05BA10 | N05BA48 |
|  |  | N05BA11 | N05BA49 |
|  |  | N05BA12 | N05BA50 |
|  |  | N05BA13 | N05BA51 |
|  |  | N05BA14 | N05BA52 |
|  |  | N05BA15 | N05BA53 |
|  |  | N05BA16 | N05BA54 |
|  |  | N05BA17 | N05BA55 |
|  |  | N05BA18 | N05BA56 |
|  |  | N05BA19 | N03AE01 |
|  |  | N05BA20 | N05CD01 |
|  |  | N05BA21 | N05CD02 |
|  |  | N05BA22 | N05CD03 |
|  |  | N05BA23 | N05CD04 |
|  |  | N05BA24 | N05CD05 |
|  |  | N05BA25 | N05CD06 |
|  |  | N05BA26 | N05CD07 |
|  |  | N05BA27 | N05CD08 |
|  |  | N05BA28 | N05CD09 |
|  |  | N05BA29 | N05CD10 |
|  |  | N05BA30 | N05CD11 |
|  |  | N05BA31 | N05CD12 |
|  |  | N05BA32 | N05CD13 |
|  |  | N05BA33 | N05CD14 |
|  |  | N05BA34 | N05CD15 |
|  |  | N05BA35 | N05CF01 |
|  |  | N05BA36 | N05CF02 |
|  |  | N05BA37 | N05CF03 |
|  |  | N05BA38 | N05CF04 |
|  |  |  | N03AE01 |

| Laxatives | | | |  | Anticholinergic agents |
| --- | --- | --- | --- | --- | --- |
| A06AA01 | A06AB36 | A06AD03 | A06AG18 |  | N04AA01 |
| A06AA02 | A06AB37 | A06AD04 | A06AG19 |  | N04AA02 |
| A06AA51 | A06AB38 | A06AD05 | A06AG20 |  | N04AA03 |
| A06AB01 | A06AB39 | A06AD06 | A06AH01 |  | N04AA04 |
| A06AB02 | A06AB40 | A06AD07 | A06AH02 |  | N04AA05 |
| A06AB03 | A06AB41 | A06AD08 | A06AH03 |  | N04AA06 |
| A06AB04 | A06AB42 | A06AD09 | A06AH04 |  | N04AA07 |
| A06AB05 | A06AB43 | A06AD10 | A06AH05 |  | N04AA08 |
| A06AB06 | A06AB44 | A06AD11 | A06AX01 |  | N04AA09 |
| A06AB07 | A06AB45 | A06AD12 | A06AX02 |  | N04AA10 |
| A06AB08 | A06AB46 | A06AD13 | A06AX03 |  | N04AA11 |
| A06AB09 | A06AB47 | A06AD14 | A06AX04 |  | N04AA12 |
| A06AB10 | A06AB48 | A06AD15 | A06AX05 |  | A01AD11 |
| A06AB11 | A06AB49 | A06AD16 | A06AX06 |  | A16AX02 |
| A06AB12 | A06AB50 | A06AD17 | A06AX07 |  |  |
| A06AB13 | A06AB51 | A06AD18 | A06AX08 |  |  |
| A06AB14 | A06AB52 | A06AD19 | A06AX09 |  |  |
| A06AB15 | A06AB53 | A06AD20 |  |  |  |
| A06AB16 | A06AB54 | A06AD21 |  |  |  |
| A06AB17 | A06AB55 | A06AD61 |  |  |  |
| A06AB18 | A06AB56 | A06AD65 |  |  |  |
| A06AB19 | A06AB57 | A06AG01 |  |  |  |
| A06AB20 | A06AB58 | A06AG02 |  |  |  |
| A06AB21 | A06AC01 | A06AG03 |  |  |  |
| A06AB22 | A06AC02 | A06AG04 |  |  |  |
| A06AB23 | A06AC03 | A06AG05 |  |  |  |
| A06AB24 | A06AC04 | A06AG06 |  |  |  |
| A06AB25 | A06AC05 | A06AG07 |  |  |  |
| A06AB26 | A06AC06 | A06AG08 |  |  |  |
| A06AB27 | A06AC07 | A06AG09 |  |  |  |
| A06AB28 | A06AC08 | A06AG10 |  |  |  |
| A06AB29 | A06AC51 | A06AG11 |  |  |  |
| A06AB30 | A06AC52 | A06AG12 |  |  |  |
| A06AB31 | A06AC53 | A06AG13 |  |  |  |
| A06AB32 | A06AC54 | A06AG14 |  |  |  |
| A06AB33 | A06AC55 | A06AG15 |  |  |  |
| A06AB34 | A06AD01 | A06AG16 |  |  |  |
| A06AB35 | A06AD02 | A06AG17 |  |  |  |

| Analgesic drugs | | | |
| --- | --- | --- | --- |
| N02AA01 | N02AX02 | N02BA75 | N02CA07 |
| N02AA02 | N02AX03 | N02BA77 | N02CA51 |
| N02AA03 | N02AX05 | N02BA79 | N02CA52 |
| N02AA04 | N02AX06 | N02BB01 | N02CA72 |
| N02AA05 | N02AJ01 | N02BB02 | N02CC01 |
| N02AA07 | N02AJ02 | N02BB03 | N02CC02 |
| N02AA08 | N02AJ03 | N02BB04 | N02CC03 |
| N02AA10 | N02AJ06 | N02BB05 | N02CC04 |
| N02AA51 | N02AJ07 | N02BB51 | N02CC05 |
| N02AA53 | N02AJ08 | N02BB52 | N02CC06 |
| N02AA55 | N02AJ09 | N02BB53 | N02CC07 |
| N02AA56 | N02AJ13 | N02BB54 | N02CX01 |
| N02AA58 | N02AJ14 | N02BB71 | N02CX02 |
| N02AA59 | N02AJ15 | N02BB72 | N02CX03 |
| N02AA79 | N02AJ17 | N02BB73 | N02CX05 |
| N02AB01 | N02AJ18 | N02BB74 | N02CX06 |
| N02AB02 | N02AJ19 | N02BE01 | N02CX07 |
| N02AB03 | N02BA01 | N02BE03 |  |
| N02AB52 | N02BA02 | N02BE04 |  |
| N02AB53 | N02BA03 | N02BE05 |  |
| N02AB72 | N02BA04 | N02BE51 |  |
| N02AB73 | N02BA05 | N02BE53 |  |
| N02AC01 | N02BA06 | N02BE54 |  |
| N02AC03 | N02BA07 | N02BE71 |  |
| N02AC04 | N02BA08 | N02BE73 |  |
| N02AC05 | N02BA09 | N02BE74 |  |
| N02AC52 | N02BA10 | N02BG02 |  |
| N02AC54 | N02BA11 | N02BG03 |  |
| N02AC74 | N02BA12 | N02BG04 |  |
| N02AC90 | N02BA14 | N02BG05 |  |
| N02AD01 | N02BA15 | N02BG06 |  |
| N02AD02 | N02BA16 | N02BG07 |  |
| N02AE01 | N02BA51 | N02BG08 |  |
| N02AE90 | N02BA55 | N02BG09 |  |
| N02AE99 | N02BA57 | N02BG10 |  |
| N02AF01 | N02BA59 | N02CA01 |  |
| N02AF02 | N02BA65 | N02CA02 |  |
| N02AX01 | N02BA71 | N02CA04 |  |
