# Supplementary material for: Impact of multi-professional medication reviews (MPMRs) on medication prescriptions in psychiatry
Source: PLoS One. 2026 Sep 21;21(9):e0358539. doi: 10.1371/journal.pone.0358539 (PMC13592593; doi:10.1371/journal.pone.0358539)
Supplement: Supplementary file 3 [file pone.0358539.s002.docx]

**S 2 Table**. **Number of molecules per class in hospital discharge prescriptions, before and after MPMR implementation.**

|  | Before MPMR  Number (%) | After one + MPMR  Number (%) |
| --- | --- | --- |
|  |  |  |
| Psychotropic drugs | | |
| 0 | 0 (0) | 0 (0) |
| 1 | 4 (3.33) | 5 (4.17) |
| 2 | 5 (4.17) | 15 (12.5) |
| 3 | 16 (13.33) | 30 (25) |
| 4+ | 95 (79.17) | 70 (58.33) |
| Benzodiazepines | | |
| 0 | 22 (18.33) | 46 (38.33) |
| 1 | 42 (35) | 37 (30.83) |
| 2 | 31 (25.83) | 27 (22.5) |
| 3 | 20 (16.67) | 8 (6.67) |
| 4+ | 5 (4.17) | 2 (1.67) |
| Antipsychotics | | |
| 0 | 2 (1.67) | 2 (1.67) |
| 1 | 23 (19.17) | 24 (20) |
| 2 | 30 (25) | 36 (30) |
| 3 | 38 (31.67) | 34 (28.33) |
| 4+ | 27 (22.50) | 24 (20) |
| Antidepressants | | |
| 0 | 96 (80) | 99 (82.5) |
| 1 | 19 (15.83) | 18 (15) |
| 2 | 4 (3.33) | 3 (2.5) |
| 3 | 1 (0.83) | 0 (0) |
| 4+ | 0 (0) | 0 (0) |
| Mood stabilizers | | |
| 0 | 85 (70.83) | 73 (60.83) |
| 1 | 28 (23.33) | 43 (35.83) |
| 2 | 6 (5) | 2 (1.67) |
| 3 | 1 (0.83) | 1 (0.83) |
| 4+ | 0 (0) | 1 (0.83) |
| Anticholinergic agents | | |
| 0 | 77 (64.17) | 112 (93.33) |
| 1 | 38 (31.67) | 8 (6.67) |
| 2 | 0 (0) | 0 (0) |
| 3 | 0 (0) | 0 (0) |
| 4+ | 0 (0) | 0 (0) |
| Laxatives | | |
| 0 | 65 (54.17) | 67 (55.83) |
| 1 | 41 (34.17) | 34 (28.33) |
| 2 | 11 (9.17) | 9 (7.5) |
| 3 | 1 (0.83) | 4 (3.33) |
| 4+ | 2 (1.67) | 6 (5) |
| Analgesic drugs | | |
| 0 | 76 (63.33) | 80 (66.67) |
| 1 | 37 (30.83) | 37 (30.83) |
| 2 | 7 (5.83) | 2 (1.67) |
| 3 | 0 (0) | 1 (0.83) |
| 4+ | 0 (0) | 0 (0) |
