# Supplementary material for: Impact of multi-professional medication reviews (MPMRs) on medication prescriptions in psychiatry
Source: PLoS One. 2026 Sep 21;21(9):e0358539. doi: 10.1371/journal.pone.0358539 (PMC13592593; doi:10.1371/journal.pone.0358539)
Supplement: Supplementary file 4 [file pone.0358539.s003.docx]

**S 3 Table**. **Mixed Linear Model (MLM) regression output regarding primary outcome.**

**Table:** Mixed Linear Model Regression: Differential of total number of molecules

| Variable | Coef. | P>\|z\| | 95 %Confidence interval |
| --- | --- | --- | --- |
| Intercept | -0.232 | 0.784 | [-1.892 ; 1.429] |
| Ward [T.1822] | -0.118 | 0.775 | [-0.927 ; 0.691] |
| Ward [T.1825] | 0.185 | 0.645 | [-0.602 ; 0.972] |
| Ward [T.1828] | -0.076 | 0.854 | [-0.889 ; 0.736] |
| Intervention (MPMR) | -0.658 | 0.001 | [-1.064 ; -0.253] |
| Age | 0.012 | 0.481 | [-0.021 ; 0.044] |
| Sex | 0.077 | 0.856 | [-0.757 ; 0.911] |
| Duration of stay | 0.001 | 0.815 | [-0.007 ; 0.009] |
| Number of MPMR | -0.003 | 0.92 | [-0.064 ; 0.058] |
