# Supplementary material for: Impact of multi-professional medication reviews (MPMRs) on medication prescriptions in psychiatry
Source: PLoS One. 2026 Sep 21;21(9):e0358539. doi: 10.1371/journal.pone.0358539 (PMC13592593; doi:10.1371/journal.pone.0358539)
Supplement: Peer review history

# Peer review: Decision Letter 0

**DOI:** 10.1371/journal.pone.0358539.r001

**Authors:** Jen Edwards

18 Jun 2026

PLOS One

Dear Dr. LEBRAT,

Thank you for submitting your manuscript to PLOS ONE. After careful consideration, we feel that it has merit but does not fully meet PLOS ONE’s publication criteria as it currently stands. Therefore, we invite you to submit a revised version of the manuscript that addresses the points raised during the review process.

Please note that we have only been able to secure a single reviewer to assess your manuscript. We are issuing a decision on your manuscript at this point to prevent further delays in the evaluation of your manuscript. Please be aware that the editor who handles your revised manuscript might find it necessary to invite additional reviewers to assess this work once the revised manuscript is submitted. However, we will aim to proceed on the basis of this single review if possible.

The reviewer has raised a number of concerns that need attention. They request the use of updated references, as well as clarification on a number of points. Could you please revise the manuscript to carefully address the concerns raised?

Please submit your revised manuscript by Aug 02 2026 11:59PM. If you will need more time than this to complete your revisions, please reply to this message or contact the journal office at plosone@plos.org. When you’re ready to submit your revision, log on to https://www.editorialmanager.com/pone/ and select the 'Submissions Needing Revision' folder to locate your manuscript file.

- A letter that responds to each point raised by the academic editor and reviewer(s). You should upload this letter as a separate file labeled 'Response to Reviewers'.
- A marked-up copy of your manuscript that highlights changes made to the original version. You should upload this as a separate file labeled 'Revised Manuscript with Track Changes'.
- An unmarked version of your revised paper without tracked changes. You should upload this as a separate file labeled 'Manuscript'.

If you would like to make changes to your financial disclosure, please include your updated statement in your cover letter. Guidelines for resubmitting your figure files are available below the reviewer comments at the end of this letter.

If applicable, we recommend that you deposit your laboratory protocols in protocols.io to enhance the reproducibility of your results. Protocols.io assigns your protocol its own identifier (DOI) so that it can be cited independently in the future. For instructions see: https://journals.plos.org/plosone/s/submission-guidelines#loc-laboratory-protocols. Additionally, PLOS ONE offers an option for publishing peer-reviewed Lab Protocol articles, which describe protocols hosted on protocols.io. Read more information on sharing protocols at https://plos.org/protocols?utm_medium=editorial-email&utm_source=authorletters&utm_campaign=protocols.

As the corresponding author, your ORCID iD is verified in the submission system and will appear in the published article. PLOS supports the use of ORCID, and we encourage all coauthors to register for an ORCID iD and use it as well. Please encourage your coauthors to verify their ORCID iD within the submission system before final acceptance, as unverified ORCID iDs will not appear in the published article. *Only*  the individual author can complete the verification step; PLOS staff *cannot*  verify ORCID iDs on behalf of authors.

We look forward to receiving your revised manuscript.

Kind regards,

Jen Edwards

Staff Editor

PLOS One

Journal requirements:

When submitting your revision, we need you to address these additional requirements.

1. Please ensure that your manuscript meets PLOS ONE's style requirements, including those for file naming. The PLOS ONE style templates can be found at

https://journals.plos.org/plosone/s/file?id=wjVg/PLOSOne_formatting_sample_main_body.pdf and

https://journals.plos.org/plosone/s/file?id=ba62/PLOSOne_formatting_sample_title_authors_affiliations.pdf

2. In the online submission form you indicate that your data is not available for proprietary reasons and have provided a contact point for accessing this data. Please note that your current contact point is a co-author on this manuscript. According to our Data Policy, the contact point must not be an author on the manuscript and must be an institutional contact, ideally not an individual. Please revise your data statement to a non-author institutional point of contact, such as a data access or ethics committee, and send this to us via return email. Please also include contact information for the third party organization, and please include the full citation of where the data can be found.

If the reviewer comments include a recommendation to cite specific previously published works, please review and evaluate these publications to determine whether they are relevant and should be cited. There is no requirement to cite these works unless the editor has indicated otherwise.

[Note: HTML markup is below. Please do not edit.]

Reviewers' comments:

Reviewer's Responses to Questions

**Comments to the Author**

1. Is the manuscript technically sound, and do the data support the conclusions?

Reviewer #1: Yes

**********

2. Has the statistical analysis been performed appropriately and rigorously?

Reviewer #1: I Don't Know

**********

3. Have the authors made all data underlying the findings in their manuscript fully available?

The PLOS Data policy

Reviewer #1: Yes

**********

4. Is the manuscript presented in an intelligible fashion and written in standard English?

Reviewer #1: Yes

**********

Reviewer #1: The paper is essentially describing the role of the MDT in delivery of pharmaceutical care, specifically assessing appropriateness of medication at discharge. The authors state that there is little known about this within psychiatry/in-patient care (lines 126-134) as the literature tends to focus on the role of pharmacy/pharmacists to conduct med reviews etc. Nevertheless, the topic has moved on considerably from the state that is suggested by the authors in the introduction. More recent and relevant references could be used (avoid anything >20yrs old)

The methodology is appropriate- perhaps call it a pre-post study and not a before and after (line 151). Line 160 should say MPMRs were implemented on 1/1/2020 instead of have been.

line 174 states that the design restricts bias but it should be acknowledged that most mental illness have variable presentation and treatment types and doses will vary throughout the lifetime so the prescribing patterns may reflect he patients current presentation and not necessarily all be due to the intervention.

More details are needed about the issue of 'sex related differences in response to psychotropic medication' (line 289)- The references suggest variance in outcomes (which is more complicated than response to medication) and/or dose response/dosages used. However, most antipsychotic meds are not prescribed according to weight (mg/kg) and therefore not impacted by sex. This statement therefore requires more detail/clarification.

The use of the term psychotropic medication as a category is misleading, esp as it is given as a separate entity from Benzo's, antipsychotopics and anticholinergics which are themselves also psychotropic meds. I suspect this category contains antidepressants/mood stabilizers and other CNS medication but this detail should be provided in the results/methods.

Numerical values should be reported with appropriate decimal places (one decimal place is often sufficient e.g SD= 20.2, 3.8) and a ZERO should also be inserted prior to the decimal point e.g 0.5, 0.05 etc.

**********

what does this mean?). If published, this will include your full peer review and any attached files.

If you choose “no”, your identity will remain anonymous but your review may still be made public.

**Do you want your identity to be public for this peer review?**  For information about this choice, including consent withdrawal, please see our Privacy Policy

Reviewer #1: No

**********

[NOTE: If reviewer comments were submitted as an attachment file, they will be attached to this email and accessible via the submission site. Please log into your account, locate the manuscript record, and check for the action link "View Attachments". If this link does not appear, there are no attachment files.]

To ensure your figures meet our technical requirements, please review our figure guidelines: https://journals.plos.org/plosone/s/figures

You may also use PLOS’s free figure tool, NAAS, to help you prepare publication quality figures: https://journals.plos.org/plosone/s/figures#loc-tools-for-figure-preparation.

NAAS will assess whether your figures meet our technical requirements by comparing each figure against our figure specifications.

# Author comment: Author response to Decision Letter 1

**DOI:** 10.1371/journal.pone.0358539.r002

24 Jun 2026

Dear Jen Edwards,

We would like to thank you and reviewer 1 for the feedback provided on our manuscript. We sincerely appreciate the time and effort invested in reviewing our work, as well as the valuable suggestions offered to improve it.

In response to the Editor’s and reviewer’s suggestions, we have revised the manuscript accordingly and are pleased to submit this revision. Below, we provide a detailed, point-by-point response to each comment. For ease of reference, the reviewers’ comments are reproduced in bold, followed by our responses. All revisions made to the manuscript are indicated with line numbers.

Point by point response to Editor:

Regarding the comment 1: “Please ensure that your manuscript meets PLOS ONE's style requirements, including those for file naming.”

Author’s response: We thank the Editor for this comment. A careful review of the manuscript has been conducted, and it has been updated to meet PLOS ONE’s style requirements.

Regarding the comment 2: “In the online submission form you indicate that your data is not available for proprietary reasons and have provided a contact point for accessing this data. Please note that your current contact point is a co-author on this manuscript. According to our Data Policy, the contact point must not be an author on the manuscript and must be an institutional contact, ideally not an individual. Please revise your data statement to a non-author institutional point of contact, such as a data access or ethics committee, and send this to us via return email. Please also include contact information for the third party organization, and please include the full citation of where the data can be found.”

Author’s response: We thank the Editor for this comment. We have revised the Data Availability Statement to provide a non-author institutional contact, in accordance with the journal's Data Policy. The institutional contact for the Ethics Committee of Centre Hospitalier Le Vinatier has been provided as the point of contact for data access (e-mail: CEREVI@ch-le-vinatier.fr). The full citation describing the data source is included in the manuscript, under the “Data availability” section.

Point by point response to Reviewer 1:

Regarding the comment 1: “The paper is essentially describing the role of the MDT in delivery of pharmaceutical care, specifically assessing appropriateness of medication at discharge. The authors state that there is little known about this within psychiatry/in-patient care (lines 126-134) as the literature tends to focus on the role of pharmacy/pharmacists to conduct med reviews etc. Nevertheless, the topic has moved on considerably from the state that is suggested by the authors in the introduction. More recent and relevant references could be used (avoid anything >20yrs old)”

Author’s response: We thank the reviewer for this relevant suggestion. We have updated the Introduction to incorporate recent literature and better reflect the current state of evidence regarding multidisciplinary medication management in psychiatric settings.

At line 117, we updated the text as follows:

“Increasing evidence supports the integration of clinical pharmacists within multidisciplinary mental health teams, where their involvement improves the identification of drug-related problems, medication reconciliation, prescribing quality, and medication safety [19, and new Ref. DOI: 10.1007/s11096-022-01409-4]”

We also tempered our wording by replacing "largely unexplored" with "less explored in the literature", to better reflect the current evidence base.

To acknowledge recent developments in the field, we added the following paragraph with new references (line 147):

“Recent evidence has expanded the literature on multidisciplinary medication management in psychiatric settings. Several studies have demonstrated that structured interdisciplinary approaches involving pharmacists improve medication safety during transitions of care and reduce medication-related problems. In a quasi-experimental study conducted in a psychogeriatric unit, interdisciplinary medication reconciliation at discharge significantly reduced reconciliation errors from 55.2% to 37% (DOI: 10.1016/j.gaceta.2025.102501). Likewise, a quality-improvement project in an inpatient psychiatry unit identified numerous prescribing errors, highlighting the value of systematic multidisciplinary review (DOI:10.9740/mhc.2024.04.097), while a pilot study in a psychiatric emergency department demonstrated significant reductions in medication discrepancies following pharmacist-supported medication reconciliation (DOI: 10.1007/s40267-021-00824-3).”

Accordingly, we have reframed the rationale for the study to acknowledge the growing body of evidence supporting multidisciplinary medication management in psychiatric settings, while clarifying that our study addresses the comparatively underexplored model of routine, ward-wide multidisciplinary medication review.

Line 164, we modified: “Given the complexity of psychiatric pharmacotherapy, effective medication management for individuals with mental disorders requires a highly specialized and collaborative approach that MPMR could bring. Evidence documenting its impact in psychiatry remains scarce.”

By:

“Nevertheless, these interventions mainly focus on medication reconciliation at transitions of care, discharge optimisation, or targeted pharmacist-led reviews. While multidisciplinary medication optimisation in psychiatric settings has been increasingly described in recent years, this ward-wide multidisciplinary medication review model, in which all prescriptions for all psychiatric inpatients are jointly reassessed during routine clinical practice, has received comparatively little attention. This distinction underpins the rationale for the present study.”

Regarding the comment 2: “The methodology is appropriate- perhaps call it a pre-post study and not a before and after (line 151).”

Author’s response: We thank the reviewer for this helpful suggestion. We have adopted the term "pre-post study" throughout the manuscript to ensure consistent terminology.

Regarding the comment 3: “Line 160 should say MPMRs were implemented on 1/1/2020 instead of have been.”

Author’s response: We thank the reviewer for this helpful suggestion. We have revised the manuscript accordingly. The sentence now reads (line 188):

“MPMRs were implemented on 1/1/2020.”

Regarding the comment 4: “line 174 states that the design restricts bias but it should be acknowledged that most mental illness have variable presentation and treatment types and doses will vary throughout the lifetime so the prescribing patterns may reflect he patients current presentation and not necessarily all be due to the intervention.”

Author’s response: We thank the reviewer for this insightful comment. We agree that, although using each patient as their own comparator reduces inter-individual variability, changes in prescribing over time may also reflect the fluctuating course of psychiatric disorders and evolving treatment needs rather than the intervention alone. We have therefore revised the Methods section (line 196) to clarify this point and have also strengthened the Limitations section to explicitly acknowledge the potential influence of changes in patients' clinical presentation over time:

The Method section now reads (line 196):

“The use of each patient’s pre-intervention stay as a comparator aimed to minimize potential confounding factors, such as inter-individual variability and differences in prescribing practices between patients. However, given the fluctuating nature of psychiatric disorders and the evolution of patients' clinical status over time, changes in prescribing patterns may also reflect variations in disease presentation, treatment response, or illness progression, rather than the effect of the intervention alone.”

The Limitation section now reads (line 481):

“First, the non-randomized pre–post design cannot fully rule out confounding factors or temporal changes in patients' clinical status. Because psychiatric disorders often have fluctuating courses and treatment requirements evolve over time, some observed changes in prescribing may reflect changes in disease severity or clinical presentation rather than the MPMR intervention itself.”

Regarding the comment 5: “More details are needed about the issue of 'sex related differences in response to psychotropic medication' (line 289)- The references suggest variance in outcomes (which is more complicated than response to medication) and/or dose response/dosages used. However, most antipsychotic meds are not prescribed according to weight (mg/kg) and therefore not impacted by sex. This statement therefore requires more detail/clarification.”

Author’s response: We thank the reviewer for this thoughtful comment. We agree that our original wording lacked precision and could be interpreted as implying that psychotropic medications are routinely prescribed according to sex or body weight. Our intention was instead to justify the inclusion of sex as a potential effect modifier in the mixed linear model, based on previous literature suggesting sex-related differences in treatment response and clinical outcomes associated with psychotropic medications. We have therefore revised the Methods section to clarify this rationale and have also replaced one of the 3 previous references with 2 more appropriate references specifically addressing sex differences in psychopharmacology (line 317):

“Because previous studies have suggested that sex may influence the clinical response to psychotropic medications and treatment outcomes in patients receiving antipsychotic therapy, sex was explored as a potential effect modifier by including an interaction term with the intervention period in the mixed linear model [32–34] (And we replaced Ref. 32 by DOI: 10.1002/wps.21155, and DOI: 10.3390/jpm16040189)”

Regarding the comment 6: “The use of the term psychotropic medication as a category is misleading, esp as it is given as a separate entity from Benzo's, antipsychotopics and anticholinergics which are themselves also psychotropic meds. I suspect this category contains antidepressants/mood stabilizers and other CNS medication but this detail should be provided in the results/methods.”

Author’s response: We thank the reviewer for this helpful comment. We agree that the presentation of the medication categories could have been clearer. Our intention was for "psychotropic medications" to represent the overall category, while benzodiazepines, antipsychotics, antidepressants, and mood stabilizers were analysed separately as psychotropic subclasses. Anticholinergic agents, laxatives, and analgesics were included as additional medication categories because of their relevance to the management of psychotropic treatment and its adverse effects. To avoid ambiguity, we have revised the Methods section to clarify this categorization and now explicitly state that the corresponding ATC codes defining each category and subclass are provided in S1 Table. We have also amended the Results section by referring to overall psychotropic medications, as well as throughout the manuscript, to distinguish this analysis from those of the individual subclasses.

Line 246, we modified:

“Secondary outcomes focused on specific drug categories to explore more targeted prescribing changes. We assessed the impact of MPMRs on the change in the number of prescriptions for the following drug classes: (i) psychotropic drugs, (ii) benzodiazepines, (iii) antipsychotics, (iv) antidepressants, (v) mood stabilizers, (vi) anticholinergic agents, (vii) laxatives and (viii) analgesic drugs.

Anticholinergic agents (vi) and laxatives (vii) were examined to evaluate the impact of MPMRs on the management of common adverse events in psychiatric settings, as these two classes are frequently used to manage psychotropic-induced side effects [27,28].

The number of prescribed benzodiazepines (ii) was used as a proxy indicator of compliance with French recommendations, given that benzodiazepine use is considered excessive in France [29,30].

The Anatomical Therapeutic Chemical (ATC) Classification System was used to identify medications belonging to each category. The full list of ATC codes used in this study is available in Supplementary File I.”

By:

“Secondary outcomes focused on changes in prescriptions for psychotropic medications overall (i), as well as for selected psychotropic subclasses analysed separately (benzodiazepines (ii), antipsychotics (iii), antidepressants (iv), and mood stabilizers (v)). We also assessed anticholinergic agents (vi), laxatives (vii), and analgesics (viii) because of their relevance to the management of psychotropic treatment and its adverse effects [27,28].

The number of prescribed benzodiazepines (ii) was used as a proxy indicator of compliance with French recommendations, given that benzodiazepine use is considered excessive in France [29,30].

The Anatomical Therapeutic Chemical (ATC) Classification System was used to identify medications belonging to each category. The ATC codes used to define each medication category and subclass are provided in S1 Table.”

In the result section, under the “secondary outcomes”, we have modified the title as follows line 382:

“Overall psychotropic medications”

Regarding the comment 7: “Numerical values should be reported with appropriate decimal places (one decimal place is often sufficient e.g SD= 20.2, 3.8) and a ZERO should also be inserted prior to the decimal point e.g 0.5, 0.05 etc.”

Author’s response: We thank the reviewer for this helpful suggestion. We have revised the numerical formatting throughout the manuscript to improve consistency and readability. Descriptive statistics are now reported using an appropriate number of decimal places (generally one decimal place), while inferential statistics (e.g., regression coefficients and p-values) are reported according to standard statistical reporting conventions. In addition, leading zeros have been added before decimal points throughout the manuscript.

We hope that these revisions adequately address all concerns and that the revised manuscript will be suitable for publication in Plos One.

Sincerely,

Matthieu Lebrat, corresponding author.

On behalf of all authors.

Attachment Submitted filename: Response to Reviewers.pdf

# Peer review: Decision Letter 1

**DOI:** 10.1371/journal.pone.0358539.r003

**Authors:** Omid Beiki

2 Sep 2026

Impact of Multi-Professional Medication Reviews (MPMRs) on medication prescriptions in psychiatry

PONE-D-26-11858R1

Dear Dr. LEBRAT,

We’re pleased to inform you that your manuscript has been judged scientifically suitable for publication and will be formally accepted for publication once it meets all outstanding technical requirements.

Within one week, you’ll receive an e-mail detailing the required amendments. When these have been addressed, you’ll receive a formal acceptance letter and your manuscript will be scheduled for publication.

An invoice will be generated when your article is formally accepted. Please note, if your institution has a publishing partnership with PLOS and your article meets the relevant criteria, all or part of your publication costs will be covered. Please make sure your user information is up-to-date by logging into Editorial Manager at Editorial Manager® and clicking the ‘Update My Information' link at the top of the page. For questions related to billing, please contact billing support.

If your institution or institutions have a press office, please notify them about your upcoming paper to help maximize its impact. If they’ll be preparing press materials, please inform our press team as soon as possible -- no later than 48 hours after receiving the formal acceptance. Your manuscript will remain under strict press embargo until 2 pm Eastern Time on the date of publication. For more information, please contact onepress@plos.org.

Kind regards,

Omid Beiki, MD, PhD, MSc

Academic Editor

PLOS One

Additional Editor Comments (optional):

Reviewers' comments:

Reviewer's Responses to Questions

**Comments to the Author**

Reviewer #2: (No Response)

**********

2. Is the manuscript technically sound, and do the data support the conclusions?

Reviewer #2: Yes

**********

3. Has the statistical analysis been performed appropriately and rigorously?

Reviewer #2: Yes

**********

4. Have the authors made all data underlying the findings in their manuscript fully available?

The PLOS Data policy

Reviewer #2: Yes

**********

5. Is the manuscript presented in an intelligible fashion and written in standard English?

Reviewer #2: Yes

**********

Reviewer #2: Dear Authors,

The authors' responses are adequate, rigorous, and conceptually well-justified. They readily accepted virtually all of the reviewers' suggestions, making substantial changes to the literature, methodological writing, statistical presentation, and clarification of variables.

The responses are well-justified, robust, and adequately address my questions and comments.

There are no serious inconsistencies or evasive answers. Acceptance of the manuscript can be recommended.

Congratulations on your work.

**********

what does this mean?). If published, this will include your full peer review and any attached files.

If you choose “no”, your identity will remain anonymous but your review may still be made public.

**Do you want your identity to be public for this peer review?**  For information about this choice, including consent withdrawal, please see our Privacy Policy

Reviewer #2: No

**********

# Editor report: Acceptance letter

**DOI:** 10.1371/journal.pone.0358539.r004

**Authors:** Omid Beiki

PONE-D-26-11858R1

PLOS One

Dear Dr. Lebrat,

I'm pleased to inform you that your manuscript has been deemed suitable for publication in PLOS One. Congratulations! Your manuscript is now being handed over to our production team.

At this stage, our production department will prepare your paper for publication. This includes ensuring the following:

* All references, tables, and figures are properly cited

* All relevant supporting information is included in the manuscript submission,

* There are no issues that prevent the paper from being properly typeset

You will receive further instructions from the production team, including instructions on how to review your proof when it is ready. Please keep in mind that we are working through a large volume of accepted articles, so please give us a few days to review your paper and let you know the next and final steps.

Lastly, if your institution or institutions have a press office, please let them know about your upcoming paper now to help maximize its impact. If they'll be preparing press materials, please inform our press team within the next 48 hours. Your manuscript will remain under strict press embargo until 2 pm Eastern Time on the date of publication. For more information, please contact onepress@plos.org.

You will receive an invoice from PLOS for your publication fee after your manuscript has reached the completed accept phase. If you receive an email requesting payment before acceptance or for any other service, this may be a phishing scheme. Learn how to identify phishing emails and protect your accounts at https://explore.plos.org/phishing.

If we can help with anything else, please email us at customercare@plos.org.

Thank you for submitting your work to PLOS One and supporting open access.

Kind regards,

PLOS One Editorial Office Staff

on behalf of

Dr. Omid Beiki

Academic Editor

PLOS One
